# Adipose-derived mesenchymal stem cell-secreted extracellular vesicles alleviate non-alcoholic fatty liver disease *via* delivering miR-223-3p

**DOI:** 10.1080/21623945.2022.2098583

**Published:** 2022-09-12

**Authors:** Qinghui Niu, Ting Wang, Zhiqiang Wang, Feng Wang, Deyu Huang, Huali Sun, Hanyun Liu

**Affiliations:** a Department of Liver Center, the Affiliated Hospital of Qingdao University, Qingdao P.R. China; b Department of Infectious Diseases, the Affiliated Hospital of Qingdao University, Qingdao P.R. China

**Keywords:** Non-alcoholic fatty liver disease, Adipose-derived mesenchymal stem cells, extracellular vesicles, MicroRNA-223-3p, E2F1, fibrosis, lipid accumulation

## Abstract

Increasing studies have identified the potential of mesenchymal stem cell-derived extracellular vesicles (MSC-EVs) in non-alcoholic fatty liver disease (NAFLD) treatment. Hence, we further focused on the potential of adipose-derived MSC (ADSC)-EVs in NAFLD by delivering miR-223-3p. The uptake of isolated ADSC-EVs by hepatocytes was assessed, and the expression of miR-223-3p in ADSC-EVs and hepatocytes was characterized. It was established that miR-223-3p, enriched in ADSC-EVs, could be delivered by ADSC-EVs into hepatocytes. Using co-culture system and gain-of-function approach, we evaluated the effect of ADSC-EVs carrying miR-223-3p on lipid accumulation and liver fibrosis in pyrrolizidine alkaloids (PA)-induced hepatocytes and a high-fat diet-induced NAFLD mouse model. Bioinformatics websites and dual-luciferase reporter gene assay were performed to determine the interactions between miR-223-3p and E2F1, which was further validated by rescue experiments. ADSC-EVs containing miR-223-3p displayed suppressive effects on lipid accumulation and liver fibrosis through E2F1 inhibition, since E2F1 was demonstrated as a target gene of miR-223-3p. The protective role of ADSC-EVs by delivering miR-223-3p was then confirmed in the mouse model. Collectively, this study elucidated that ADSC-EVs delayed the progression NAFLD through the delivery of anti-fibrotic miR-223-3p and subsequent E2F1 suppression, which may suggest miR-223-3p-loaded ADSC-EVs to be a potential therapeutic approach for NAFLD.

## Introduction

Non-alcoholic fatty liver disease (NAFLD), a chronic disease with a global prevalence of over 25% [1], is categorized as non-alcoholic fatty liver (NAFL) or non-alcoholic steatohepatitis (NASH) [2,3]. NAFLD is characterized by accumulation of fat, which further leads to liver inflammation or even progresses to NASH, fibrosis, cirrhosis or liver cancer [4,5]. It is interesting to note that mesenchymal stem cell-derived extracellular vesicles (MSC-EVs) have been associated with promising therapeutic potential for liver diseases such as liver fibrosis and liver damage [6]. However, the specific mechanistic actions of MSC-EVs on NAFLD remain to be established.

MSCs are adult stromal cells with multilineage differentiation potentials, and MSC-EVs can transfer components including messenger RNAs (mRNAs), microRNAs (miRNAs, miRs), lipids, or organelles to participate in disease progression [7–10]. Among them, adipose-derived MSCs (ADSCs) have gained widespread attention in recent years for their multifaceted capabilities, abundance and easy isolation [11]. Notably, restoration of ADSCs might be a potential strategy against obesity and the associated disorders including NAFLD [12], due to its function on repressing inflammatory infiltration, abrogating fibrosis, and restoring liver tissues [13].

Of interest, EVs are small lipid membrane vesicles that can be secreted from multiple kinds of cells, including ADSCs [14,15]. EVs are known as potential circulating biomarkers for various diseases due to their involvement in cell-cell communication in the body [16,17]. Emerging evidence has also reported that EVs have a pivotal role to play in the pathogenesis of metabolic liver diseases, such as NAFLD, through shuttling bioactive components, especially miRNAs, as cargos to modulate metabolic alterations in recipient cells [18–20].

Intriguingly, emerging data has indicated the involvement of granulocyte-enriched miR-223 [21] in chronic hepatic injuries such as NASH as well as in acute hepatitis [22]. Further, miR-223-3p, as a miR-223 analogue, has been recognized to ameliorate acute and chronic hepatitis through repressing the activation of the NLRP3 inflammasome [23]. miR-223-3p delivery *via* EVs derived from bone marrow MSCs has been illustrated to alleviate autoimmune liver disease by attenuating inflammatory responses [24]. A prior study has attributed the alleviatory effect of miR-223-3p against inflammation diseases to targeted inhibition of E2F transcription factor 1 (E2F1) [25]. Furthermore, E2F1 has been suggested to modulate cholestatic liver fibrosis, highlighting a promise as fibrogenic marker [26].

Hence, it can be hypothesized that miR-223-3p delivered by ADSC-EVs may affect the progression of NAFLD through controlling E2F1. The present study was designed to test this hypothesis and to provide a better understanding of the molecular mechanism associated with NAFLD.

## Methods

### Ethics statement

The current study was performed under the approval of the Animal Ethics Committee of the Affiliated Hospital of Qingdao University. All animal experiments were performed in strict accordance with the recommendations in the Guide for the Care and Use of Laboratory Animals of the National Institutes of Health.

### In silico analysis

The expression profiles of miR-223-3p in EVs were retrieved from the EVmiRNA database. The downstream genes of miR-223-3p were predicted through microRNA, mirDIP and TargetScan databases, respectively, which were intersected to obtain the intersected genes. The intersected genes were then subjected to interaction analysis using the STRING database. A gene interaction network was constructed using cytoscape v3.7.1 software, and the core value of each gene was calculated.

### Isolation of ADSCs

Eight-week-old mice were euthanized and then soaked in 75% ethanol for 5 min. The inguinal adipose tissues of mice were separated in the ultra-clean workbench, the blood vessels, lymph nodes and fascia around adipose tissues were removed as much as possible, and adipose tissues were washed twice with PBS. The adipose tissues were cut into small pieces and transferred to a 50 mL centrifuge tube which was added with an equal volume of 0.1% type I collagenase later. The centrifuge tube was placed on a constant temperature shaker at 37°C and shaken at 250 rpm for 60 min for digestion. Then the tube was added with DMEM/F12 medium containing 10% FBS to terminate the digestion. After the remaining tissue mass was filtered through a cell sieve, the digested tissues were centrifuged (1500 rpm) for 5 min. After resuspension, the tissues were seeded into a 25 cm culture flask, and cultured. After 48 h, the medium was renewed. When the cell density reached 90%, cells were detached with 0.25% trypsin containing ethylene diamine tetraacetic acid for passage. ADSCs at passage 2 or above with good growth status were selected for subsequent experiments.

### Identification of ADSCs

After the cell confluence of ADSCs at passage 3 reached 80%. For osteogenic induction, ADSCs were cultured in osteogenic induction medium (Gibco) for 21 days, and the cultured cells were subsequently stained with Alizarin Red S to detect the deposition of calcified matrix. ADSCs were cultured in adipogenic differentiation medium to identify the adipogenic differentiation ability. After 14 days, oil red O staining [27] was carried out to characterize the morphological changes under an inverted phase contrast microscope. The expression of ADSC surface marker antigens CD49d, CD90, CD105, CD34, CD45, and CD106 were identified by flow cytometry.

### Isolation and identification of ADSCs-EVs

FBS was ultracentrifuged at 100,000 g at 4°C for 7 h, and the supernatant was taken to prepare a EV-depleted complete medium. ADSCs at passage 3 were cultured for 48 h in the EV-depleted complete medium, and then the supernatant culture medium was collected. EVs were separated and extracted by density gradient centrifugation method. Specifically, the supernatant was subjected to 10-min centrifugation (300 g) and another 20-min one (3000 g) at 4°C, and the precipitation was discarded. After centrifugation at 100,000 g for 70 min, precipitation was collected, resuspended with PBS twice and finally filtered by a 0.22 μm filter. The obtained ADSCs-EVs were stored at −80°C for later use. Surface markers of ADSCs-EVs were detected by Western blot analysis. The following antibodies were used, rabbit anti-heat shock protein 70 (Hsp70) (ab79852, Abcam, Cambridge, UK), rabbit anti-tumour susceptibility 101 (TSG101) (ab125011, Abcam), rabbit anti-CD81 (ab109201, Abcam), rabbit anti-Calnexin (ab10286, Abcam). ADSCs-DMEM precipitation served as a negative control (NC). A transmission electron microscope (TEM) was used to observe the morphology of ADSC-EVs, and a Tecnai^TM^ Spirit (T12) TEM (HiLLsboro, Oregon) was adopted to capture images of ADSC-EVs. Nanoparticle tracking analysis (NTA) was utilized to determine the particle size and concentration using NanoSight NS300 (Malvern Panalytical, Worcestershire, UK).

### Uptake of PKH67-labelled EVs by hepatocytes

EV suspension (100 μL) was mixed with 1 mL of diluted PKH67 (4 μL/mL, Sigma-Aldrich, St. Louis, MO). EVs were re-extracted and stained green by PKH67. The nuclei of NCTC1469 cells were stained blue by DAPI (Sigma-Aldrich). PKH67-labelled EVs were incubated with NCTC1469 cells under 5% CO_2_ and 37°C for 12 h. Images were captured using a Zeiss LSM 780 confocal microscope (Zeiss, Jena, Germany).

### Construction of lentiviral vector

After double-enzyme digestion of the pLenti lentiviral backbone vector, miR-223-3p with a designed double-enzyme site was combined with the pLenti vector and then transferred to DH5α cells, followed by amplification in E. coli. Constructed lentiviral vectors were collected by plasmid sequencing. 293 T cells were incubated in a 10-cm plate, with 3 μg pLenti-miR-223-3p, 1 μg pCMV-VSV-G, and 3 μg pCMV-Delta8.9 added to each well. Next, transfection was performed utilizing Lipofectamine 3000 reagent (Invitrogen). After 24 h of transfection, cells were transferred to fresh medium for 48-h incubation, and the cell supernatant containing the virus was then collected and filtered with a 0.45-μm filter. Harvested lentiviruses were stored at −80°C.

### Cell culture and transduction

A NCTC1469 cell line (normal mouse hepatocytes) and a human embryonic kidney HEK293T cell line were purchased from American Type Culture Collection (CCL9.1, ATCC, Manassas, VA). Cells were routinely cultured in DMEM medium. Upon cell confluence reaching 90%, the cell monolayer was detached with 1 mL of 0.25% trypsin for 3–4 min. Then 3 mL of complete medium was added to the cells to terminate the digestion and suspend the cells. Cells were incubated for 24 h with or without palmitic acid (PA) for following experiment. The presence of PA could induce lipid accumulation in hepatocytes.

ADSCs were transduced with lentiviral vectors containing miR-223-3p mimic or miR-223-3p inhibitor or the corresponding negative control (NC), referred to as LV-miR-223-3p mimic, LV-miR-223-3p inhibitor, LV-NC and LV-inhibitor (Table S1). One day prior to transduction, ADSCs were seeded into 75 cm^2^ culture flasks at a concentration of 1 × 10^6^ cells/mL. When the cell fusion reached 50% – 70%, the ADSCs in flask were cultured with the above-mentioned LVs (purchased from GenePharma, Suzhou, Jiangsu, China). After stably transduced cell lines were obtained, ADSC-EVs were extracted. NCTC1469 cells were transduced with LV-sh-E2F1 or LV-oe-E2F1 before co-culture with EVs isolated from the ADSCs transduced with LV-miR-223-3p mimic or LV-miR-223-3p inhibitor.

### Establishment of a mouse model of NAFLD

C57BL/6 J female mice aged 6 weeks old (SLAC Laboratory Animal Co. Ltd., Shanghai, China) were fed with a chow diet (carbohydrates accounted for 62.3% of total calories, fat 12.5%, and protein 24.3%) or high-fat diet (HFD) (D12492, carbohydrates accounted for 32.6% of total calories, fat 51.0%, and protein 16.4%) for 8 weeks.

### Characterization of mouse model of NAFLD

We verified the successful establishment of the NAFLD model through the biochemical analysis [28] of serum alanine aminotransferase (ALT) and aspartate aminotransferase (AST) (kits from BioVision, Minneapolis, MN) as well as triglyceride (TG) and total cholesterol (TC) in liver tissues (kits from Applygen, Beijing, China), and also through oil red O staining, HE staining, hydroxyproline detection and Masson staining in the liver tissue (Fig. S1A-H), by which levels of serum ALT and AST and liver TG and TC as well as lipid accumulation and fibrosis were all confirmed to be increased in the mouse models. At the same time, we measured the body weight of the mice every week, as shown in Fig. S2.

### Experimental protocols in vivo

EVs isolated from the ADSCs transduced with LV-mimic NC, LV-miR-223-3p mimic, LV-inhibitor NC and LV-miR-223-3p inhibitor, namely, EVs-mimic NC, EVs-miR-223-3p mimic, EVs-inhibitor NC, and EVs-miR-223-3p inhibitor, respectively (100 μg/100 μL) or equal volume of PBS were injected into the tail vein of mice twice a week starting from the second week of diet. Further, 10 μL of 10^8^ TU/mL LV-sh-E2F1, LV-oe-E2F1, or LV-sh-NC was injected intraperitoneally into mice fed with an HFD. After injection, mice were kept in the animal experiment centre. Specific experimental groups are as follows: control (chow diet, only injected with PBS), NAFLD (HFD, only injected with PBS), EVs-mimic NC (HFD, injected with EVs-mimic NC), EVs-miR-223-3p mimic (HFD, injected with EVs-miR-223-3p mimic), EVs-inhibitor NC + sh-NC (HFD, injected with EVs-inhibitor NC and LV-sh-NC), EVs-miR-223-3p inhibitor + sh-NC (HFD, injected with EVs-miR-223-3p inhibitor and LV-sh-NC), and EVs-miR-223-3p inhibitor + sh-E2F1 (HFD, injected with EVs-miR-223-3p inhibitor and LV-sh-E2F1). All animals were euthanatized at the 8^th^ week, blood samples and liver tissues were collected.

### Histopathological analysis

The liver tissues of mice were fixed in 10% formalin, paraffinized, sliced and subjected to haematoxylin and eosin (H&E) staining. The paraffin-embedded liver was frozen using a cryostat and cut into 4-µm sections, fixed in 4% formalin, and then stained with oil red O/60% isopropanol solution (Thermo Fisher Scientific, Waltham, MA), and counterstained with haematoxylin, followed by observation utilizing a Zeiss Axioplan 2 upright microscope (Zeiss) [28]. In terms of Masson staining, sections were stained with iron haematoxylin, eosin acid fuchsin dye solution, and counterstained with aniline blue dye solution, followed by observation under a Zeiss Axioplan 2 upright microscope [29].

### Nile red staining

The liquid accumulation in NCTC1469 cells was detected with lipophilic dye Nile Red (Sigma-Aldrich). Briefly, NCTC1469 cells were fixed with 4% PFA for 10 min, and then incubated with 1 mg/L Nile red solution in PBS at 37°C for 20 min. Ten cells were fixed with Gold anti-fading reagent containing DAPI (Invitrogen, Carlsbad, California) and examined under a fluorescence microscope.

### Liver collagen content

Liver sections were stained with Alizarin Red. Liver hydroxyproline was determined by a hydroxyproline colorimetric assay kit (BioVision, Milpitas, CA) to quantify liver collagen content, which reflected the liver fibrosis. The hydrolysed liver tissues were neutralized with sodium hydroxide, added with Ehrlich reagent for colour development, and the optical density (OD) value at 570 nm wavelength was measured.

### Western blot analysis

Liver tissue homogenate or NCTC1469 cells were lysed using RIPA lysis buffer (Santa Cruz Biotechnology, CA) and centrifuged at 14,000 g and 4°C for 30 min. The protein concentration in the supernatant was determined using a detergent compatible assay (Dc protein assay, Bio-Rad Laboratories, Shanghai, China). The protein sample (50 μg) was separated by 10% sodium dodecyl sulphate-polyacrylamide gel electrophoresis (SDS-PAGE), and then transferred to a polyvinylidene fluoride (PVDF) membrane (Millipore Corporation, Billerica, MA). Then, the membrane was blocked with 5% BSA at room temperature for 1 h, and then incubated with primary antibodies α-smooth muscle actin (α-SMA, 1: 1000, ab32575, Abcam), collagen type I alpha 1 [COL1A1, rabbit mAb #72026, cell signalling technology (CST), Danvers, MA], transforming growth factor β1 (TGF-β1, 1: 1000, ab215715, Abcam), anti-E2F1 (ab112580, Abcam), and rabbit anti- GAPDH (1: 2000, ab181602, Abcam) overnight at 4°C. The membrane was then incubated with horseradish peroxidase (HRP)-conjugated goat anti-rabbit immunoglobulin G (IgG) (1: 1000, ab205718, Abcam) or goat anti-mouse IgG at room temperature (1: 1000, ab205719, Abcam) for 1 h. The protein bands were visualized with an ECL Plus chemiluminescence reagent kit (Amersham Biosciences, Arlington Heights, IL). Image J software (National Institutes of Health, Bethesda, MD) was used to quantify the grey value of blots, as normalized to GAPDH.

### RNA quantification

Total RNA was extracted from mouse liver tissues or cells using Trizol reagent (15,596,026, Invitrogen, Carlsbad, CA), and the concentration and purity of total RNA were measured utilizing Nanodrop ND-1000 instrument (Thermo Fisher Scientific). For mRNA detection, complementary DNA (cDNA) was synthesized using the reverse transcription kit (RR047A, Takara, Japan). For miRNA detection, cDNA was obtained utilizing the miRNA First Strand cDNA Synthesis (Tailing Reaction) Kit (B532451-0010, Sangon, Shanghai, China). The expression of miR-223-3p was determined by RT-qPCR by means of TaqMan microRNA assays (Applied Biosystems) and ABI PRISM 7300 RT-PCR system (Applied Biosystems). Other genes were measured utilizing SYBR Green PCR Master Mix reagents (Applied Biosystems) and ABI PRISM 7900 Sequence Detection System (Applied Biosystems). Primers were synthesized by Takara (Table S2). The relative expression level was normalized to GAPDH or snRNA U6 expression and was calculated using the 2^−ΔΔCt^ method.

### Dual-luciferase reporter gene assay

A binding site was predicted to exist between the 3’-untranslated region (3’UTR) of E2F1 and miR-223-3p by TargetScan database. Wild type (WT) and mutant type (MUT) vectors were constructed by amplifying E2F1-3’-UTR sequence containing miR-223-3p binding site and sequence with mutated binding site using PCR technology. These sequences were then cloned into pMIR-Report vector (Promega, Madison, WI) to generate Luc-pMIR-E2F1-3’-UTR plasmid (E2F1-WT) or Luc-pMIR-E2F1-mut-3’-UTR plasmid (E2F1-MUT). Next, 293 T cells were cultured on a 24-well plate and transfected with miR-223-3p mimic and its corresponding NC, and then transfected for 48 h with luciferase plasmids E2F1-WT or E2F1-MUT utilizing Lipofectamine 2000. Based on the Dual-Luciferase Reporter assay system (Promega), the relative luciferase activity was calculated by firefly/renilla luciferase activity.

### Statistical analysis

All experimental data were analysed using SPSS 21.0 statistical software (IBM Corp, Armonk, NY). Measurement data were all summarized by mean ± standard deviation. The comparison between two groups was conducted by unpaired *t* test. Data comparisons among multiple groups were performed using one-way analysis of variance (ANOVA) with Tukey’s post hoc test. *p* < 0.05 was considered statistically significant.

## Results

### MiR-223-3p is enriched in ADSCs-derived EVs and the EVs can be taken up by mouse NCTC1469 cells

**Figure 1.**
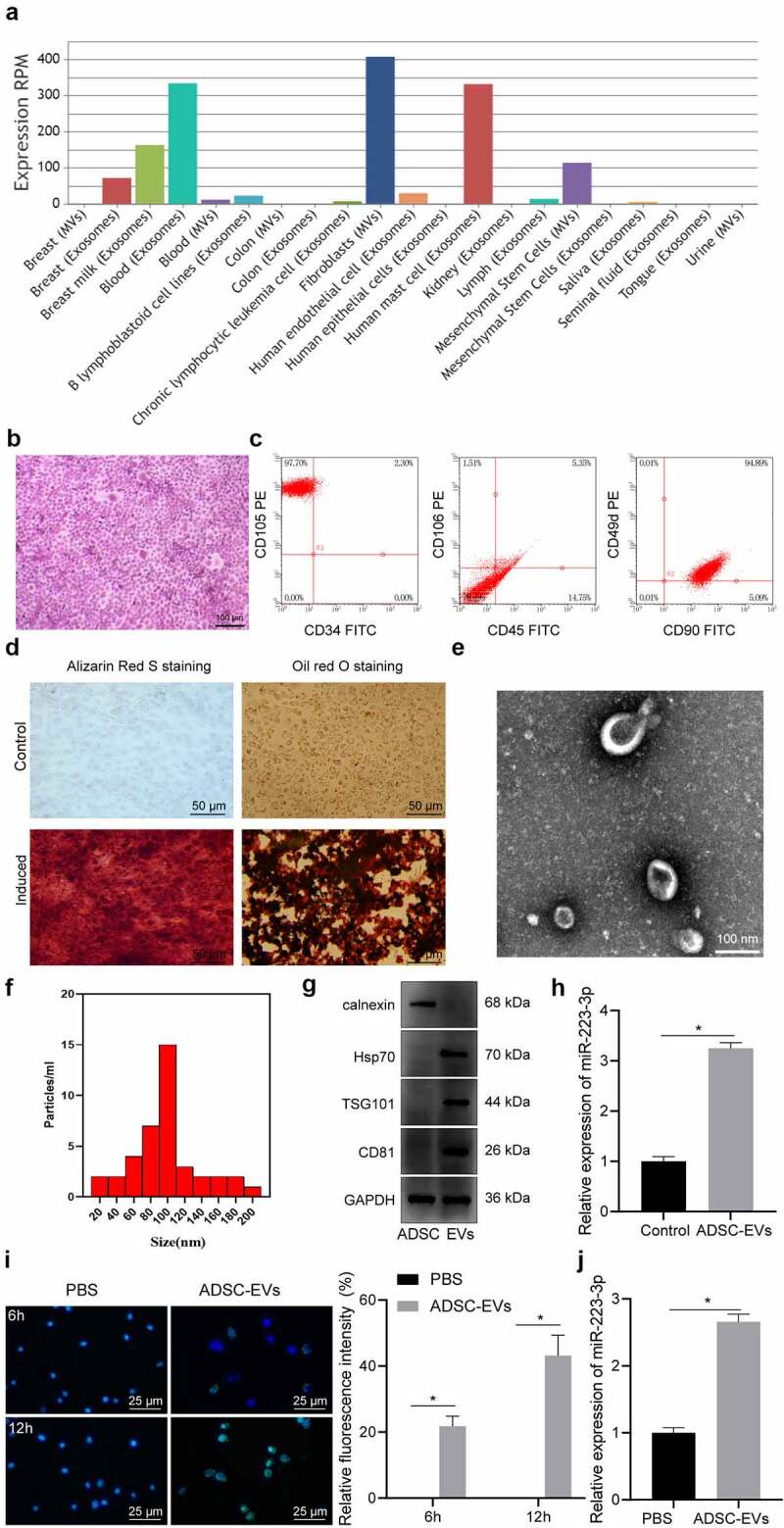
miR-223-3p in ADSCs was delivered to NCTC1469 mouse hepatocytes through EVs. a, The expression of miR-223-3p in EVs from different sources obtained from EVmiRNA database, wherein the x-axis indicates the type of EVs, and the y-axis indicates the expression value. b, ADSCs observed by inverted phase contrast microscope after H&E staining. c, The expression of surface markers CD49d, CD90, CD105, CD34, CD45, and CD106 in isolated ADSCs detected by flow cytometry. d, ADSCs stained with Alizarin Red S and oil red O of osteogenic and adipogenic differentiation. e, ADSC-EVs observed under a TEM. f, The size and concentration of ADSC-EVs detected by NTA. g, The expression of EV surface markers Hsp70, TSG101, and CD81 and endoplasmic reticulum marker Calnexin determined by Western blot analysis. h, The expression of miR-223-3p in ADSC-EVs determined by RT-qPCR. i, The uptake of ADSC-EVs by NCTC1469 cells at 6 h and 12 h observed by immunofluorescence staining, with EVs labelled by PKH67 in green and nuclei stained with DAPI in blue. j, The expression of miR-223-3p in NCTC1469 cells co-cultured with ADSC-EVs determined by RT-qPCR. Measurement data are expressed as mean ± standard deviation, * *p* < 0.05. Independent sample *t* test was used to analyse the data between two groups, and the cell experiment was repeated three times.

According to the results of EVmiRNA database, we identified the presence of miR-223-3p in EVs derived from MSCs (Figure 1a). Therefore, we tried to clarify whether ADSCs could deliver miR-223-3p to hepatocytes through EVs to participate in the regulation of NAFLD. First, mouse ADSCs were isolated (Figure 1b). Flow cytometry exhibited that CD49d, CD90, and CD105 were highly expressed in the isolated ADSCs, while CD34, CD45 and CD106 were not expressed (Figure 1c).

As presented in Figure 1d, compared with ADSCs untreated with induction medium, ADSCs induced by differentiation medium showed the potential for osteogenic and adipogenic differentiation. The isolated ADSC-EVs were cup-shaped (Figure 1e), with an average diameter of 95.8 ± 1.2 nm (figure 1f). The isolated EVs showed expression of the marker proteins Hsp70, TSG101, and CD81, but not calnexin (Figure 1g), which validated that ADSC-EVs were successfully isolated. Subsequently, we revealed that, compared with normal hepatocytes, miR-223-3p was highly expressed in ADSC-EVs (Figure 1h).

Next, NCTC1469 cells were co-cultured with fluorescent PKH67-labelled ADSC-EVs and the uptake of ADSC-EVs by hepatocytes gradually increased over time (Figure 1i). MiR-223-3p expression in NCTC1469 cells was notably increased after co-culture with ADSC-EVs (Figure 1j).

Together, miR-223-3p presents a high expression in EVs derived from mouse ADSCs, and the miR-223-3p-harboured ADSC-EVs could be taken up by NCTC1469 cells.

### ADSC-EVs deliver miR-223-3p to reduce PA-induced lipid accumulation and fibrosis in NCTC1469 cells

To further examine whether ADSC-EVs carrying miR-223-3p could participate in NAFLD, ADSCs were transduced with LV-miR-223-3p mimic, and then EVs (EVs-mimic NC and EVs-miR-223-3p mimic) were extracted. MiR-233-3p expression in EVs derived from miR-233-3p overexpression ADSCs was validated to be up-regulated (Fig. S3).

**Figure 2.**
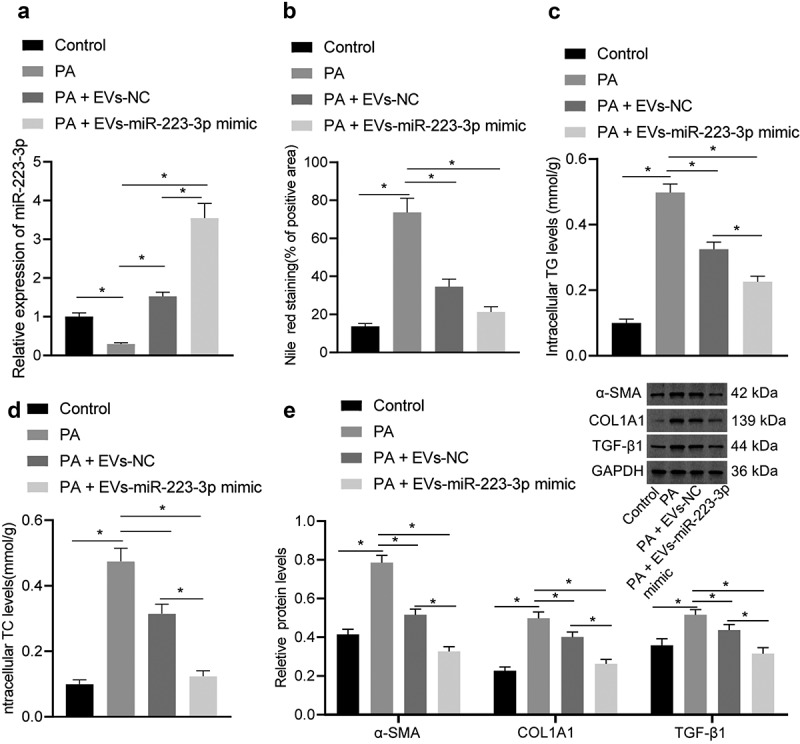
ADSC-EVs containing miR-223-3p attenuate pyrrolizidine alkaloids (PA)-induced lipid accumulation and fibrosis in NCTC1469 cells. a, The expression of miR-223-3p in NCTC1469 cells after PA induction alone or together with EV co-culture determined by RT-qPCR. b, The lipid deposition in NCTC1469 cells after PA induction alone or together with EV co-culture determined by Nile red staining. c, Triglyceride (TG) content after PA induction alone or together with EV co-culture. d, Total cholesterol (TC) content after PA induction alone or together with EV co-culture. e, The level of liver fibrosis markers α-SMA, COL1A1, TGF-β1 after PA induction alone or together with EV co-culture determined by Western blot analysis. Measurement data are expressed as mean ± standard deviation, * *p* < 0.05. Data comparison among multiple groups was analysed by one-way ANOVA followed by Tukey’s post hoc test, and the cell experiment was repeated three times.

Subsequently, the EVs were co-cultured with PA-treated NCTC1469 cells. PA induction reduced miR-223-3p expression and increased the lipid deposition as well as TG and TC contents in NCTC1469 cells. However, further co-culture with either EVs-mimic NC or EVs-miR-223-3p mimic led to an increase of miR-223-3p expression, but reductions in lipid deposition, TG and TC contents; and the aforementioned effects were more obvious in response to EVs-miR-223-3p mimic relative to EVs-mimic NC (Figure 2a-d, Fig. S4A).

In addition, Western blot assay revealed that the expression of liver fibrosis marker proteins (α-SMA, COL1A1 and TGF-β1) increased notably in cells after the induction of PA, which was repressed after the co-culture with either EVs-mimic NC or EVs-miR-223-3p mimic, and the latter exerted a stronger repressive effect versus the former (Figure 2e).

Above results suggested that ADSC-EVs played an inhibiting role in PA-induced lipid accumulation and fibrosis in NCTC1469 cells by delivering miR-223-3p.

### ADSC-EVs deliver miR-223-3p to attenuate lipid accumulation and fibrosis in NAFLD mice

**Figure 3.**
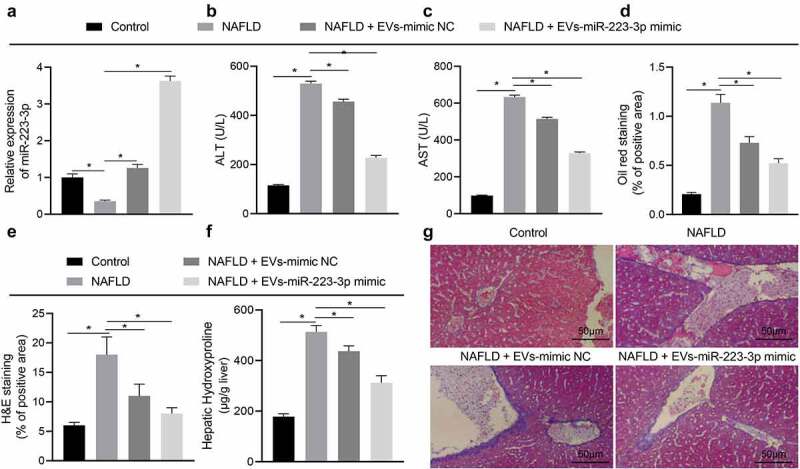
ADSC-EVs containing miR-223-3p reduce lipid accumulation and fibrosis in NAFLD mice. a, The expression of miR-223-3p in the liver tissues of NAFLD mice with/without the presence of miR-223-3p overexpression, determined by RT-qPCR. b, Alanine aminotransferase (ALT) content in serum of NAFLD mice with/without the presence of miR-223-3p overexpression. c, Aspartate aminotransferase (AST) content in serum of NAFLD mice with/without the presence of miR-223-3p overexpression. d, Lipid accumulation in liver tissues of NAFLD mice with/without the presence of miR-223-3p overexpression, detected by oil red O staining. e, H&E staining of liver tissues of NAFLD mice with/without the presence of miR-223-3p overexpression. f, The level of hydroxyproline in liver tissues of NAFLD mice with/without the presence of miR-223-3p overexpression. g, Severity of liver fibrosis in NAFLD mice with/without the presence of miR-223-3p overexpression detected by Masson staining. Measurement data are expressed as mean ± standard deviation, * *p* < 0.05. Data comparison among multiple groups was analysed by one-way ANOVA followed by Tukey’s post hoc test, n = 10.

Next, a NAFLD mouse model was established (Figs. S1-2) and injected with EVs-miR-223-3p mimic. Relative to control mice, miR-223-3p expression was reduced in the liver tissues of NAFLD mice and then rescued in response to injection of EVs-mimic NC or EVs-miR-223-3p mimic (Figure 3a). The ELISA suggested that ALT and AST levels were up-regulated in serum of NAFLD mice, and the up-regulation was reversed in response to additional treatment by EVs-mimic NC or EVs-miR-223-3p mimic; and EVs-miR-223-3p mimic led to a more obvious reversion relative to EVs-mimic NC (Figure 3b-c).

The results of oil red O staining and H&E staining showed lipid accumulation in the liver tissues of NAFLD mice. However, the treatment of EVs-mimic NC or EVs-miR-223-3p mimic could suppress the liver lipid accumulation in NAFLD mice, and EVs-miR-223-3p mimic exerted a more obvious effect (Figure 3d-e, Fig. S5A-B). Subsequently, the hydroxyproline level was increased in the liver tissues of NAFLD mice compared with control mice, which indicated the development of liver fibrosis, while EVs-mimic NC and EVs-miR-223-3p mimic treatment could reduce the hydroxyproline level, and EVs-miR-223-3p mimic showed a more remarkable effect; and the change in hydroxyproline level was consistent with that in severity of liver fibrosis, as reflected by Masson staining (figure 3f, 3).

To sum up, ADSC-EVs relieved NAFLD by repressing lipid accumulation and fibrosis in mice *via* delivering miR-223-3p.

### miR-223-3p targets and negatively regulates E2F1 expression

**Figure 4.**
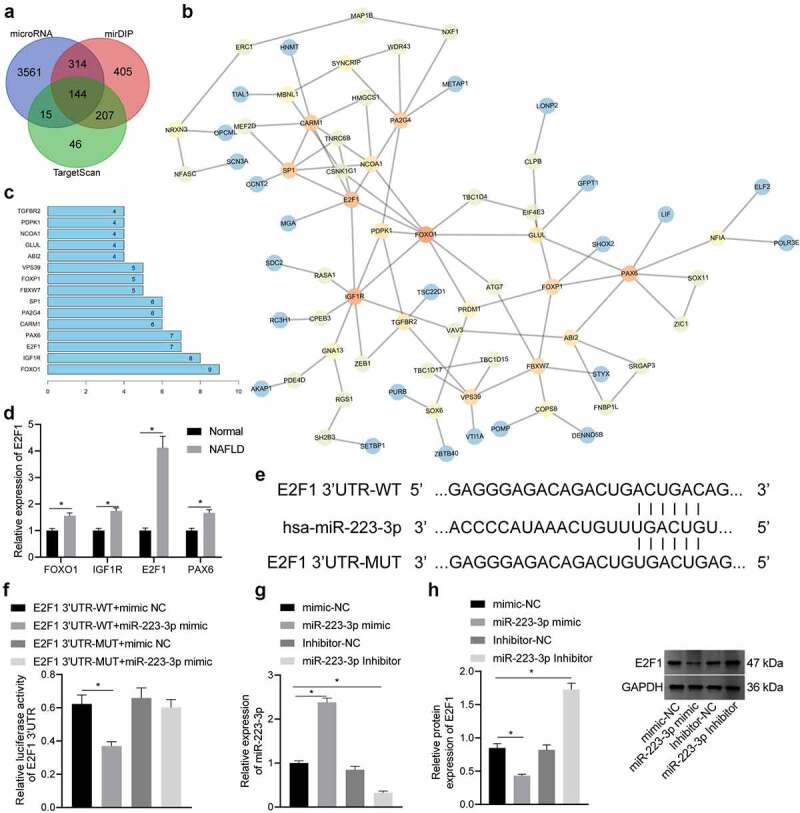
E2F1 is targeted and negatively regulated by miR-223-3p. a, Prediction results of downstream gene of miR-223-3p, wherein the three circles in the panel represent the prediction results of the miR-223-3p downstream genes from the three databases, and the middle part represents the intersection of the three sets of data. b, The interaction analysis of candidate genes. Each circle represents a gene, and the line between the circles indicates an interaction relationship between genes. The darker colour indicates higher core value. c, The core degree of the candidate genes, wherein the x-axis indicates the core degree value, and the y-axis indicates the name of the top 15 genes with the highest core degree value. d, The expression of FOXO1, IGF1R, E2F1 and PAX6 in normal liver and NAFLD liver tissues determined by RT-qPCR. e, The binding site between miR-223-3p and E2F1. f, The binding of miR-223-3p and E2F1 verified by dual-luciferase report gene assay. g, The expression of miR-223-3p in NCTC1469 cells after transduction determined by RT-qPCR. h, The protein level of E2F1 in NCTC1469 cells after transduction determined by Western blot analysis. Measurement data are expressed as mean ± standard deviation, * *p* < 0.05. Data comparison between two groups was analysed by independent t test, and comparison among multiple groups was analysed by one-way ANOVA followed by Tukey’s post hoc test, and the cell experiment was repeated three times.

To identify the downstream regulatory mechanism of miR-223-3p, we adopted microRNA database, mirDIP database and TargetScan database to predict the downstream genes of miR-223-3p, where 144 candidate genes were obtained in the intersection (Figure 4a). A gene interaction network diagram was plotted, and the core value of each gene was calculated (Figure 4b). It was revealed that FOXO1, IGF1R, E2F1 and PAX6 had the highest core values (Figure 4c), among which E2F1 was then observed by RT-qPCR to be the most differentially expressed one in NAFLD liver tissues (Figure 4d).

According to the Starbase database, E2F1 and miR-223-3p exhibited potential binding site (Figure 4e), which was validated by a dual-luciferase reporter gene assay. Results revealed that the luciferase activity of the E2F1-WT co-transfected with miR-223-3p mimic was inhibited dramatically, while the luciferase activity of E2F1-MUT co-transfected with miR-223-3p mimic did not change notably (figure 4f).

Subsequently, NCTC1469 cells were transduced with LV-miR-223-3p mimic, LV-miR-223-3p inhibitor and their NCs (LV-mimic-NC, LV-inhibitor-NC), and RT-qPCR results displayed that miR-223-3p expression was upregulated by LV-miR-223-3p mimic in NCTC1469 cells but suppressed by LV-miR-223-3p inhibitor, which validated the transduction efficiency of miR-223-3p (Figure 4g). As measured by Western blot analysis, E2F1 level was decreased by the transduction of LV-miR-223-3p mimic, but was elevated after the transduction of LV-miR-223-3p inhibitor (Figure 4h).

**Figure 5.**
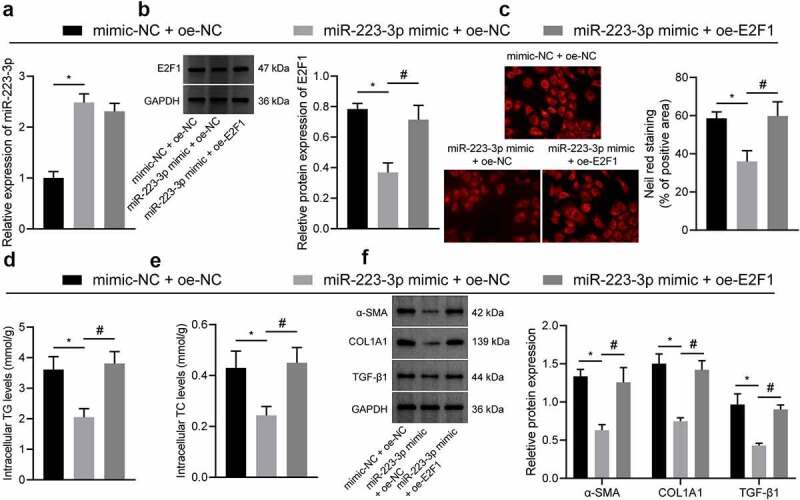
E2F1 overexpression reversed the inhibiting effects of miR-223-3p on lipid accumulating and fibrosis in hepatocyte. a, qRT-PCR measurement of the expression of miR-223-3p in cells treated with miR-223-3p mimic alone or in combination with oe-E2F1. b, Western blot measurement of the expression of E2F1 in cells treated with miR-223-3p mimic alone or in combination with oe-E2F1. c, Lipid deposition determined by Nile red staining in cells treated with miR-223-3p mimic alone or in combination with oe-E2F1. d, TC content in cells treated with miR-223-3p mimic alone or in combination with oe-E2F1. e, TG content in cells treated with miR-223-3p mimic alone or in combination with oe-E2F1. f, Western blot measurement of the protein expression of α-SMA, Coliα1 and TGF-β1 in cells treated with miR-223-3p mimic alone or in combination with oe-E2F1. Measurement data are expressed as mean ± standard deviation. * *p* < 0.05. Comparison among multiple groups was analysed by one-way ANOVA followed by Tukey’s post hoc test. The cell experiment was repeated three times.

**Figure 6.**
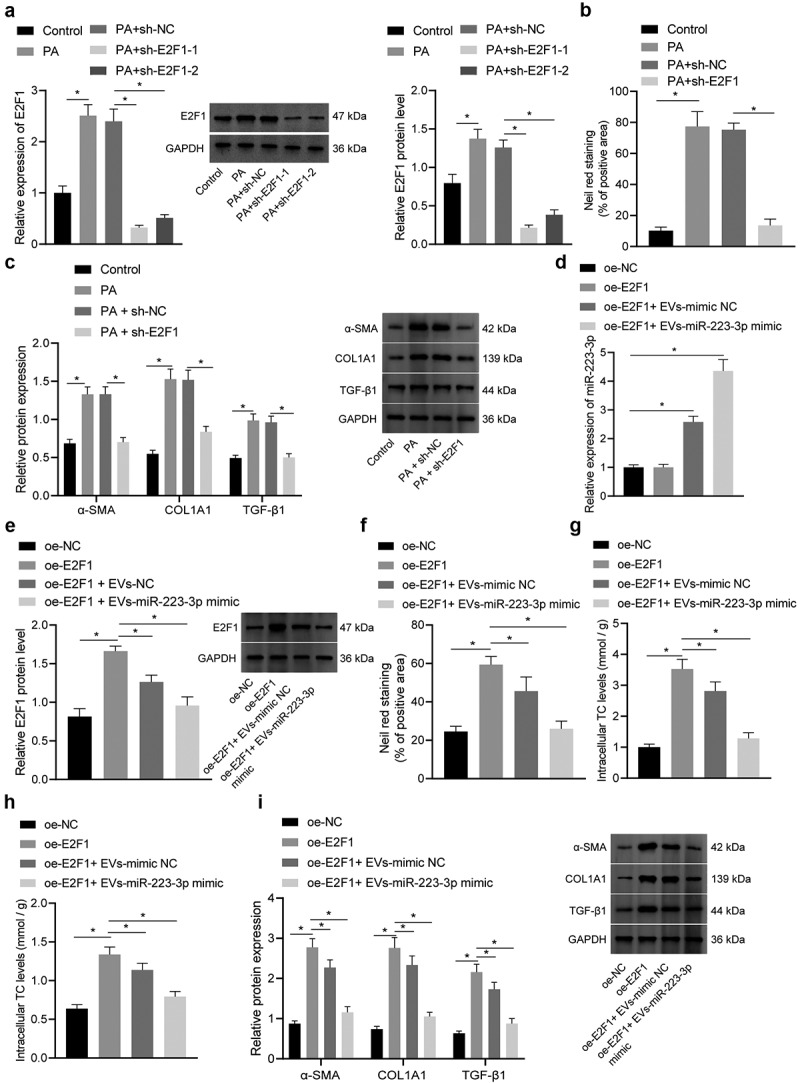
ADSC-EVs containing miR-223-3p inhibits E2F1 to reduce pyrrolizidine alkaloids (PA)-induced lipid accumulation and fibrosis *in vitro*. a, The expression of E2F1 in NCTC1469 cells after transduction and P) induction determined by RT-qPCR. b, Intracellular lipid deposition after transduction and PA induction determined by Nile red staining. c, The expression of liver fibrosis markers α-SMA, COL1A1 and TGF-β1 after transduction and PA induction determined by RT-qPCR. d, The expression of miR-223-3p in NCTC1469 cells after transduction or EV co-culture determined by RT-qPCR. e, The expression of E2F1 in NCTC1469 cells after transduction or EV co-culture determined by RT-qPCR. f, Lipid deposition determined by Nile red staining. g, TG content in NCTC1469 cells after transduction or EV co-culture. h, TC content in NCTC1469 cells after transduction or EV co-culture. i, Protein expression of liver fibrosis markers α-SMA, COL1A1, and TGF-β1 in NCTC1469 cells after transduction or EV co-culture determined by Western blot analysis. Measurement data are expressed as mean ± standard deviation, * *p* < 0.05. Comparison among multiple groups was analysed by one-way ANOVA followed by Tukey’s post hoc test, and the cell experiment was repeated three times.

Furthermore, we conducted a rescue assay to investigate the role of E2F1 in the mechanism by which miR-223-3p modulated the lipid accumulation and fibrosis. PA-treated NCTC1469 cells were treated with miR-223-3p mimic alone or in combination with E2F1 restoration. As result, miR-223-3p-induced decreases in hepatocyte lipid accumulation and fibrosis were reversed in response to additional E2F1 restoration (Figure 5 and 6).

Therefore, miR-223-3p could target and negatively regulate E2F1, and E2F1 up-regulative may abrogate the inhibiting effects of miR-223-3p on lipid accumulation and fibrosis in NAFLD.

***Promotive effects of E2F1 on cellular lipid accumulation and fibrosis can be reversed by ADSC-EVs containing miR-223-3p***

**Figure 7.**
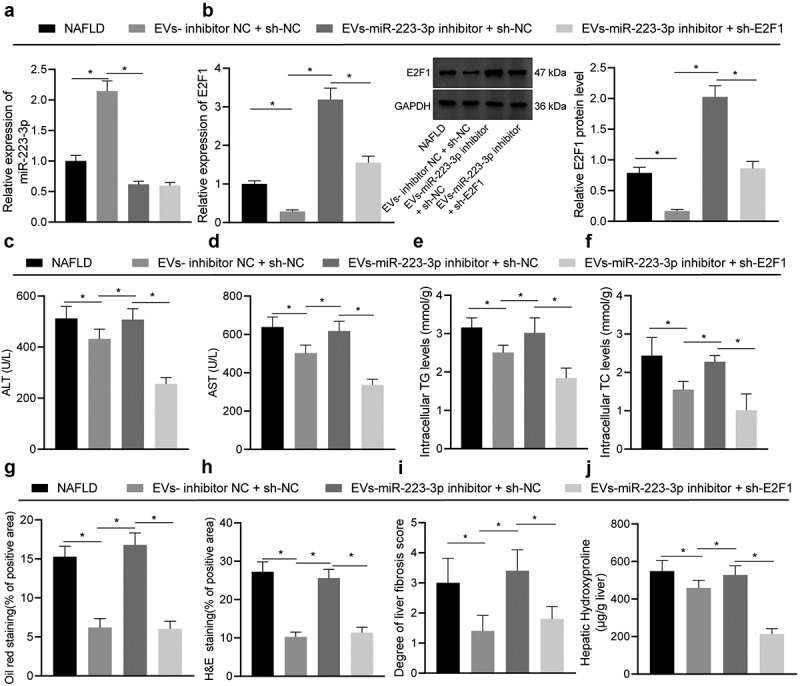
ADSC-EVs containing miR-223-3p inhibit E2F1 to reduce lipid accumulation and liver fibrosis *in vivo*. NAFLD mice were injected with EVs-miR-223-3p inhibitor alone or combined with LV-sh-E2F1. a, The expression of miR-223-3p in mouse liver tissues determined by RT-qPCR. b, The expression of E2F1 in mouse liver tissues determined by RT-qPCR (left) and Western blot analysis (right). c, Alanine aminotransferase (ALT) content in serum of NAFLD mice. d, Aspartate aminotransferase (AST) content in serum of NAFLD mice. e, Serum triglyceride (TG) level of NAFLD mice. f, Serum total cholesterol (TC) level of NAFLD mice. g, Lipid accumulation in liver tissues of NAFLD mice observed by oil red O staining. h, Alterations of liver tissues of NAFLD mice observed after H&E staining. i, Severity of liver fibrosis assessed by Masson staining. j, The level of liver hydroxyproline in liver tissues of NAFLD mice. Measurement data are expressed as mean ± standard deviation, * *p* < 0.05. Comparison among multiple groups was analysed by one-way ANOVA followed by Tukey’s post hoc test, n = 10.

To further explore the specific role of E2F1 in NAFLD, NCTC1469 cells were transduced with LV-sh-E2F1 and subjected to PA induction. RT-qPCR and Western blot assay displayed that E2F1 expression was reduced in NCTC1469 cells transduced with sh-E2F1-1 or shE2F1-2, and the knockdown effect of sh-E2F1-1 was superior (Figure 7a). Thus, sh-E2F1-1 (sh-E2F1) was used for subsequent experiments.

Nile red staining exhibited that lipid deposition was enhanced (Figure 7b, Fig. S4B). However, transduction with LV-sh-E2F1 reduced the expression of E2F1, accompanied with suppressed lipid deposition. In addition, the protein expression of liver fibrosis markers α-SMA, COL1A1 and TGF-β1 was elevated in NCTC1469 cells after PA induction, which was obviously reduced when E2F1 was knocked down (Figure 7c). Hence, E2F1 knockdown could repress lipid accumulation and fibrosis.

To investigate whether E2F1-mediated lipid accumulation and fibrosis could be reversed by miR-223-3p in ADSC-EVs, we subsequently transduced NCTC1469 cells with LV-oe-E2F1, and then treated with ADSC-EVs carrying miR-223-3p. RT-qPCR results demonstrated that LV-oe-E2F1 increased the expression of E2F1 potently without affecting miR-223-3p expression. However, the expression of miR-223-3p was upregulated while E2F1 was downregulated after treatment with EVs-mimic NC or EVs-miR-223-3p mimic (Figure 6d-e).

Subsequently, lipid deposition and the TG and TC content were all increased by overexpression of E2F1, and the increase was reversed when E2F1 overexpression was combined with EVs-mimic NC or EVs-miR-223-3p mimic (figure 6f-h, Fig. S4C). In addition, overexpression of E2F1 increased the expression of liver fibrosis markers, which was abrogated by the further treatment of EVs-mimic NC or EVs-miR-223-3p mimic (Figure 6i).

According to abovementioned results, the promoting effect of E2F1 on *in vitro* lipid accumulation and fibrosis could be reversed by miR-223-3p delivered by ADSC-EVs.

***ADSC-EVs alleviate NAFLD by reducing lipid accumulation and liver fibrosis in vivo through miR-223-3p-mediated E2F1 inhibition***

Next, the regulatory mechanism *in vivo* was verified using NAFLD mice injected with EVs-miR-223-3p inhibitor and/or LV-sh-E2F1. Initially, RT-qPCR results revealed that miR-223-3p expression was increased while E2F1 expression was decreased by EVs-inhibitor NC + LV-sh-NC in NAFLD mice. EVs-miR-223-3p inhibitor repressed expression of miR-223-3p and increased E2F1 level in NAFLD mice. Compared with NAFLD mice injected with EVs-miR-223-3p inhibitor + LV-sh-NC, the expression of miR-223-3p showed no obvious change while E2F1 was notably reduced in response to EVs-miR-223-3p inhibitor + LV-sh-E2F1 (Figure 7a).

Subsequently, ALT and AST levels in the serum of NAFLD mice were reduced by treatment with EVs-inhibitor-NC + LV-sh-NC but rescued in response to EVs-miR-223-3p inhibitor, whereas additional E2F1 silencing further reduced ALT and AST levels (Figure 7c). Consistently, increases were also observed in serum levels of TG and TC in NAFLD mice treated with EVs-miR-223-3p inhibitor alone relative to the NC group, and these increases were negated in the presence of EVs-miR-223-3p inhibitor + LV-sh-E2F1 (Figure 7e).

Besides, the lipid content in the liver tissues of the NAFLD mice after the treatment of EVs-inhibitor NC + LV-sh-NC was reduced, which was rescued after miR-223-3p inhibition in the NAFLD mice, and further E2F1 knockdown reduced the lipid content (Figure 7g-h, Fig. S5C-D). In the liver tissues of NAFLD mice, EVs-inhibitor NC + LV-sh-NC treatment attenuated fibrosis and decreased the hydroxyproline levels. However, both of them were observed to be enhanced by miR-223-3p inhibition in the NAFLD mice. Further E2F1 knockdown reversed the promoting effect of miR-223-3p inhibition alone on fibrosis and the level of hydroxyproline in liver tissues (Figure 7i-j, Fig. S5E).

Taken together, ADSC-EVs deliver miR-223-3p to negatively regulate E2F1 expression, and then reduce lipid accumulation and liver fibrosis in NAFLD mice; accordingly, miR-223-3p-loaded ADSC-EVs and E2F1 knockdown were both demonstrated to ameliorate NAFLD.

## Discussion

In our current work, we further delineated that ADSC-EVs containing miR-223-3p attenuated the lipid accumulation and liver fibrosis by repressing E2F1, whereby alleviating NAFLD. Thus, our study reveals a new mechanistic understanding of ADSC-EVs in NAFLD. More importantly and practically, this cellular communication by EVs, which connect ADSCs and hepatocytes, may provide novel options for preventive therapies in the future and may facilitate the development of personalized diagnostics and therapeutics for NAFLD.

Our initial finding indicated that miR-223-3p presented a high expression in ADSC-EVs and miR-223-3p transmitted by ADSC-EVs repressed the lipid accumulation and fibrosis in hepatocytes. Consistent with our finding, another study also revealed that miR-223-3p was enriched in MSC-EVs [30]. miR-223 was recently identified as a promising diagnostic biomarker for diverse liver diseases such as hepatitis virus infections, liver injury induced by alcohol or drug, NAFLD, cirrhosis, and liver cancer [22]. Consistently, miR-223-3p was also found to be delivered by bone marrow MSC-derived EVs, which further protect liver injury in autoimmune hepatitis [31]. Besides, a more recent finding uncovered that ADSCs served as an inhibitor of NAFLD progression by releasing anti-fibrotic miR-122 [32]. Thus, the delivery of miR-223-3p by ADSC-EVs might hold anti-fibrotic and hepatoprotective potential in NAFLD.

Moreover, ADSC-EVs carrying miR-223-3p were experimentally confirmed to confer hepatoprotection *in vitro*, corresponding to reduced contents of TG and TC as well as the expression of liver fibrosis markers α-SMA, COL1A1 and TGF-β1. TG and TC are a vital pair of hallmarks of NAFLD that indicate aberrant hepatic lipid accumulation [33–35]. α-SMA, COL1A1 and TGF-β1 are all crucial biomarkers for liver fibrosis [36–38], while their downregulation is a sign of liver fibrosis recovery [39]. Our work also highlighted that miR-223-3p from ADSC-EVs also reduced the ALT and AST levels, and hydroxyproline level *in vivo*, suggesting restoration of liver functions [40] and amelioration of NAFLD [41,42].

In the subsequent analysis, miR-223-3p was verified to negatively regulate E2F1 by binding to its 3’UTR. Consistently, E2F1 was recognized to be repressed by miR-222-3p and thus implicated in the pathogenesis of NASH associated liver carcinogenesis [43]. Our study also demonstrated that E2F1 silencing inhibited lipid accumulation and fibrosis. According to a prior study, E2F1 depletion was observed to exert a suppressing role on glycolysis and lipogenesis of hepatocytes and further contributes to the delay of NAFLD development [44]. In addition, it was also confirmed that E2F1 was a target gene regulated by miR-223, representing a potential target against inflammation diseases [25]. E2F1 was identified as a fibrogenic gene and its knockdown led to suppression of biliary fibrosis [26].

Our *in vivo* study substantiated that ADSC-EVs alleviated NAFLD by reducing lipid accumulation and liver fibrosis through miR-223-3p-mediated E2F1 inhibition. In relation to this, the liver has been recognized as a primary site of EV uptake after intravenous administration [45]. EV uptake by most cell types has been attributed to endocytosis, and various cell types in the liver, due to the complex microenvironment, may present with differentiated potential in regard of EV uptake [46]. A prior study indicated that EVs efficiently delivered miR-155 mimic or inhibitor to liver macrophages and hepatocytes both *in vitro* and *in vivo* [47]. More recently, cholangiocyte-derived exosomes were found to mainly target hepatic stellate cells (HSCs), HSC-derived fibroblasts, Kupffer cells and hepatocytes [48]. Herein, further studies are merit to explore which types of hepatocytes were mostly affected by ADSC-EVs delivering miR-223-3p *in vivo*.

## Conclusion

**Figure 8.**
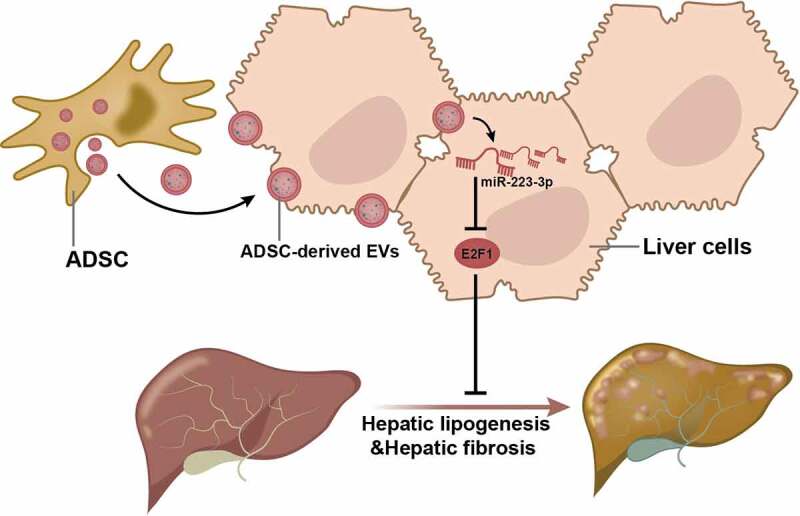
Schematic diagram shows that ADSC-EVs delayed the progression NAFLD through the delivery of anti-fibrotic miR-223-3p and subsequent E2F1 suppression.

In summary, this study provided evidence suggesting that miR-223-3p delivered by ADSC-EVs downregulated the expression of E2F1 to further attenuate the lipid accumulation and fibrosis, which ultimately relieved NAFLD (Figure 8). Nevertheless, the mechanisms mediated by E2F1 still require more in-depth investigations for a comprehensive understanding, and future trials based on the purity and dosages of EVs are necessary to achieve more efficient therapeutic function.

## Supplementary Material

Supplemental Material

## Data Availability

The datasets generated and/or analysed during the current study are available in the manuscript and supplementary materials.
