# Supplementary material for: Adipose-derived mesenchymal stem cell-secreted extracellular vesicles alleviate non-alcoholic fatty liver disease via delivering miR-223-3p
Source: Adipocyte. 2022 Sep 12;11(1):572–87. doi: 10.1080/21623945.2022.2098583 (PMC9481107; doi:10.1080/21623945.2022.2098583)
Supplement: Supplemental Material [file KADI_A_2098583_SM8241.zip › supplementary/Table S1.docx]

**Table S1**

Primer sequences of miR-233-3p mimic/inhibitor

| Gene | Sequence (5’-3’) |
| --- | --- |
| miR-223-3p mimic (hsa/mmu) | GCGCGTGTCAGTTTGTCAAAT |
| miR-223-3p inhibitor (hsa/mmu) | ATTTGACAAACTGACACGCGC |
| mimic NC (hsa/mmu) | GCGATGGACCCATTGAGAGC |
| inhibitor NC (hsa/mmu) | CGATCGACCAGATTACGTAC |

Note: miR-223-3p, microRNA-223-3p; NC, negative control
