# Supplementary material for: Adipose-derived mesenchymal stem cell-secreted extracellular vesicles alleviate non-alcoholic fatty liver disease via delivering miR-223-3p
Source: Adipocyte. 2022 Sep 12;11(1):572–87. doi: 10.1080/21623945.2022.2098583 (PMC9481107; doi:10.1080/21623945.2022.2098583)
Supplement: Supplemental Material [file KADI_A_2098583_SM8241.zip › supplementary/Table S2.docx]

**Table S2**

Primer sequences of RT-qPCR

| Gene | Sequence (5’-3’) |
| --- | --- |
| miR-223-3p (hsa/mmu) | Forward: GCGCGTGTCAGTTTGTCAAAT |
|  | Reverse: Universal reverse primer |
| U6 (hsa/mmu) | Forward: AGAGAAGATTAGCATGGCCCCTG |
|  | Reverse: Universal reverse primer |
| E2F1 (mmu) | Forward: ATTTGGCGCGTAAAAGTGGC |
|  | Reverse: CATAGATGCGCCGTTTCTGC |
| E2F1 (hsa) | Forward: ACAAGGCCCGATCGATGTTT |
|  | Reverse: CTGCAGAGACAAGGTGAGCA |
| GAPDH (hsa) | Forward: CCATGGGGAAGGTGAAGGTC |
|  | Reverse: AGTGATGGCATGGACTGTGG |
| GAPDH (mmu) | Forward: CCCTTAAGAGGGATGCTGCC |
|  | Reverse: ACTGTGCCGTTGAATTTGCC |
| IGF1R (mmu) | Forward: GACTTCGGACCAGTCTCGC |
|  | Reverse: TTGCAAACGCAGAGATGCAG |
| PAX6 (mmu) | Forward: CACCAGACTCACCTGACACC |
|  | Reverse: TCACTCCGCTGTGACTGTTC |
| FOXO1 (mmu) | Forward: TGTACAGCGCATAGCACCAA |
|  | Reverse: CCGATGGACGGAATGAGAGG |

Note: RT-qPCR, reverse transcription quantitative polymerase chain reaction; miR-223-3p, microRNA-223-3p; E2F1, E2F transcription factor 1; GAPDH, glyceraldehyde-3-phosphate dehydrogenase; IGF1R, insulin like growth factor 1 receptor; PAX6, paired box 6; FOXO1, forkhead box O1.
