# Supplementary figures and images for: Adipose-derived mesenchymal stem cell-secreted extracellular vesicles alleviate non-alcoholic fatty liver disease via delivering miR-223-3p
Source: Adipocyte. 2022 Sep 12;11(1):572–87. doi: 10.1080/21623945.2022.2098583 (PMC9481107; doi:10.1080/21623945.2022.2098583)

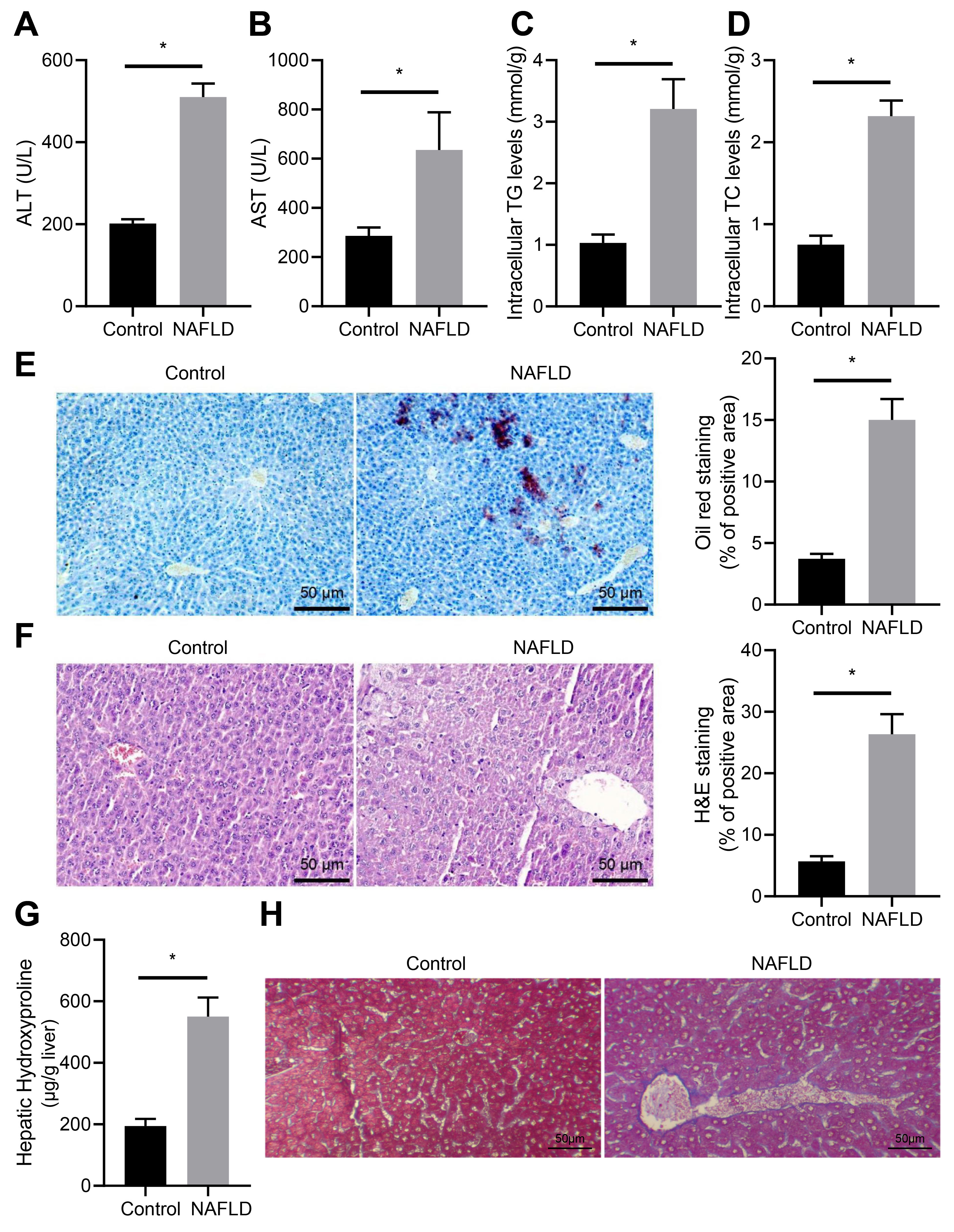

Supplement: Supplemental Material [file KADI_A_2098583_SM8241.zip › supplementary/Figure S1.jpg]

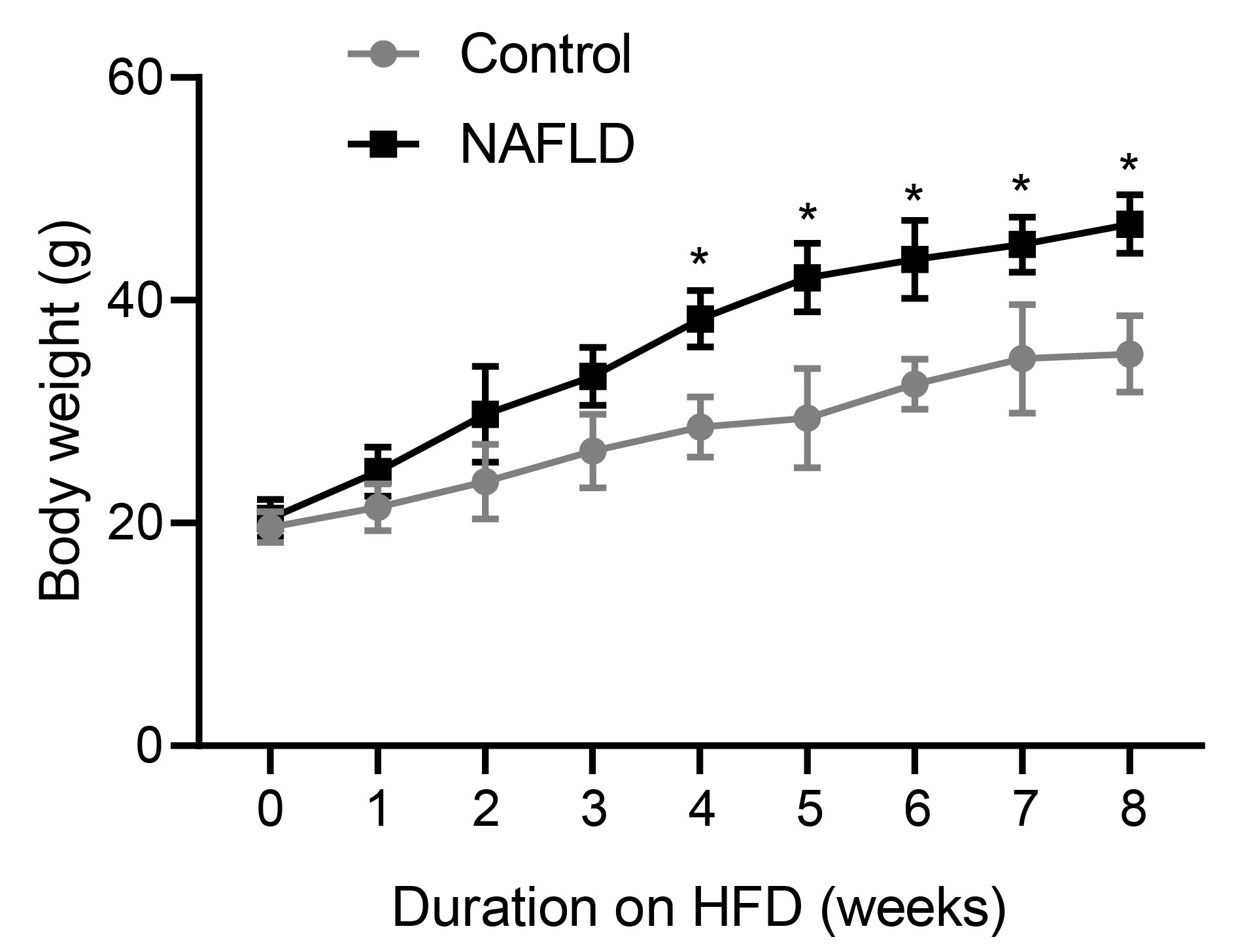

Supplement: Supplemental Material [file KADI_A_2098583_SM8241.zip › supplementary/Figure S2.jpg]

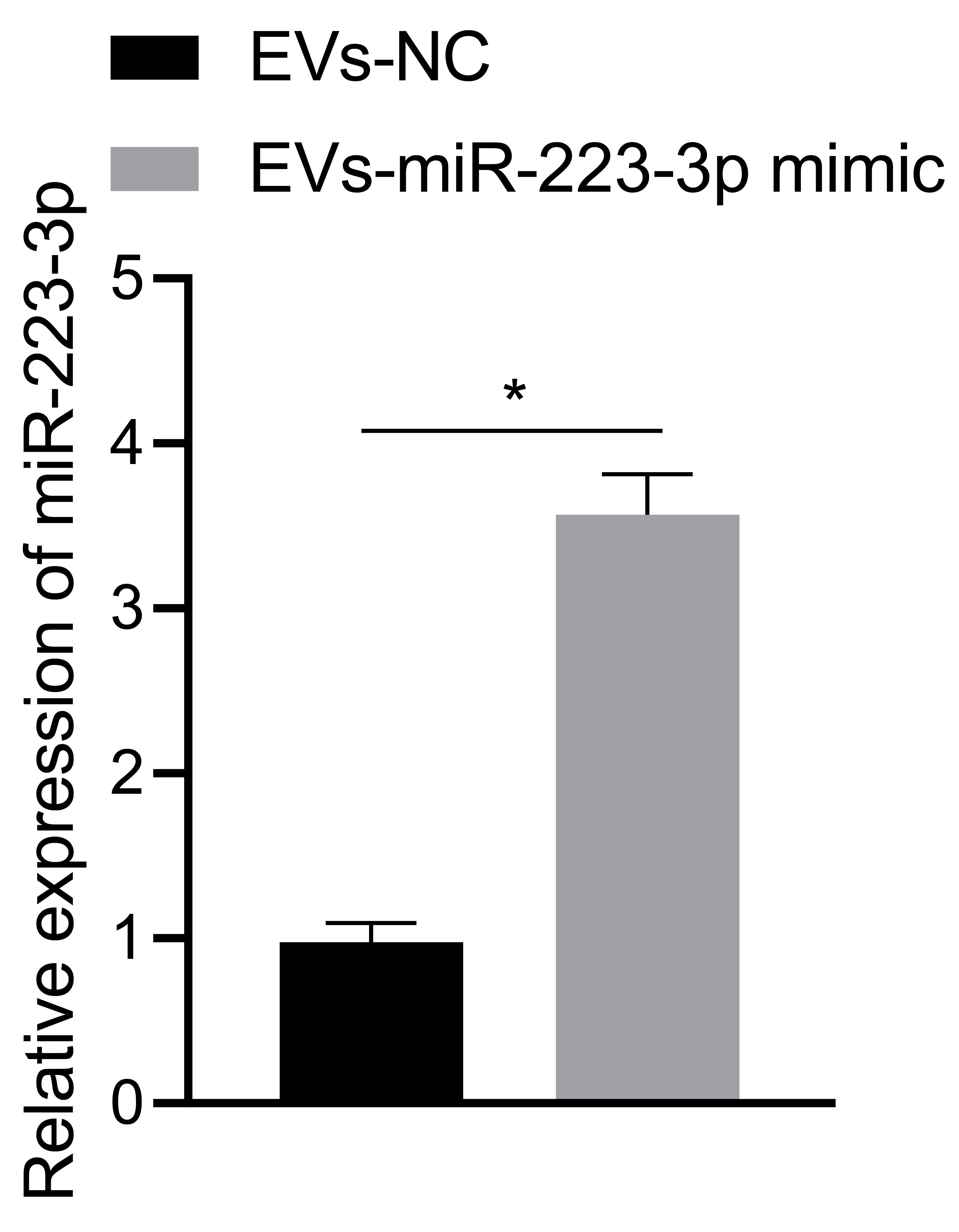

Supplement: Supplemental Material [file KADI_A_2098583_SM8241.zip › supplementary/Figure S3.jpg]

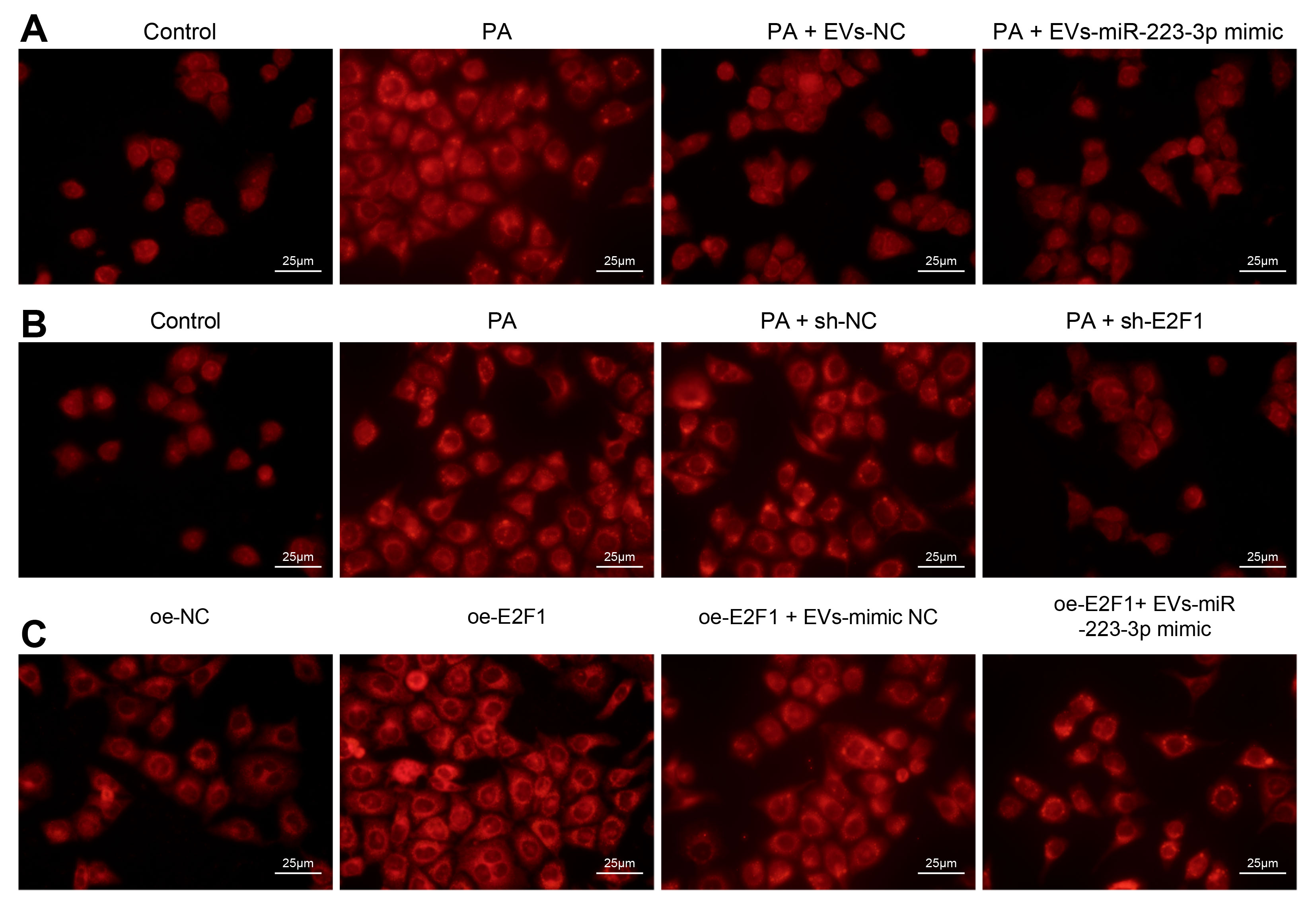

Supplement: Supplemental Material [file KADI_A_2098583_SM8241.zip › supplementary/Figure S4.jpg]

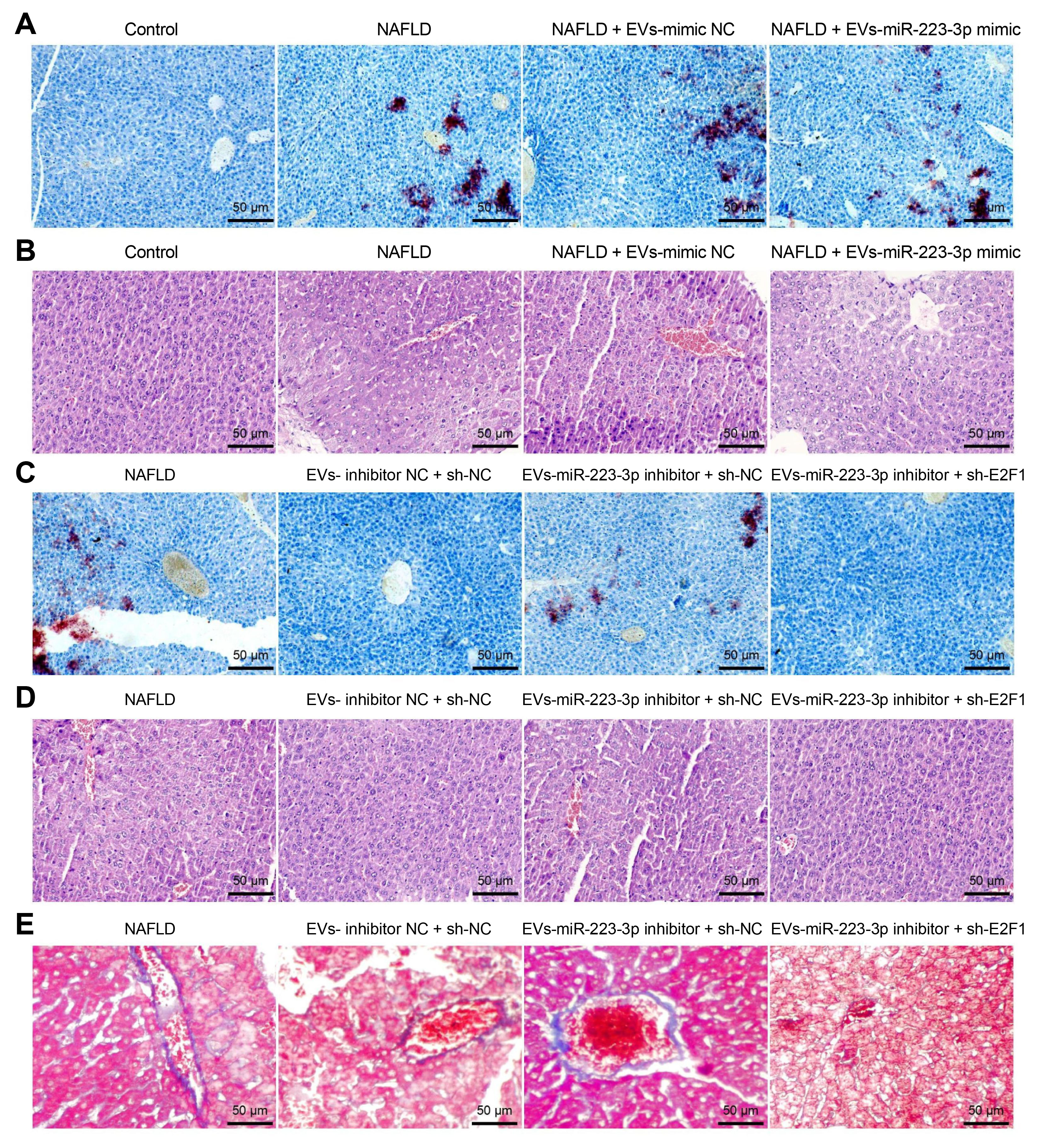

Supplement: Supplemental Material [file KADI_A_2098583_SM8241.zip › supplementary/Figure S5.jpg]
